# Discovery of endogenous retroviruses in the genome of the red panda (*Ailurus fulgens*)

**DOI:** 10.1128/spectrum.00024-25

**Published:** 2025-07-15

**Authors:** Yingying Bao, Lichen Mao, Alfred Ndjekadom, Wenhui Shi, Chenglin Zhou, Juan Xu, Xiaochun Wang, Yuwei Liu, Shixing Yang, Likai Ji, Tongling Shan, Wen Zhang, Quan Shen

**Affiliations:** 1 Department of Laboratory Medicine, School of Medicine, Jiangsu University, Zhenjiang, China; 2 Clinical Laboratory Center, The Affiliated Taizhou People’s Hospital of Nanjing Medical University, Taizhou, China; 3 Shanghai Veterinary Research Institute, Chinese Academy of Agricultural Sciences, Shanghai, China; Penn State College of Medicine, Hershey, Pennsylvania, USA

**Keywords:** red panda, *Ailurus fulgens*, endogenous retrovirus, genome, phylogenetic analysis, evolution

## Abstract

Endogenous retroviruses (ERVs) are viral genomes that originate from the integration of exogenous retroviruses into host germ cells and can be stably inherited across generations. Despite being mostly incomplete and silent, several ERVs have been observed to play potential pathological roles in numerous diseases, including multiple sclerosis, rheumatoid arthritis, and notably, cancer. Research has revealed the presence of ERVs in the genomes of nearly all mammals. However, the distribution and diversity of these retroviral genes in the endangered red panda (*Ailurus fulgens*) remain largely unexplored. This study aimed to identify and characterize ERV sequences in the red panda genome using bioinformatic methods. The results revealed the presence of 11 ERV sequences in the red panda genome, among which 6 contain complete viral sequences with long terminal repeats (LTRs), indicating proviral structures. Furthermore, phylogenetic analysis based on crucial retroviral protein structures (GAG, POL, and ENV) showed that nine ERVs belonged to the Gammaretrovirus genus and two to the Betaretrovirus genus, suggesting stable clustering within their respective taxonomic groups. Notably, the red panda ERVs clustered with retroviral sequences from various hosts, including Muridae, Felidae, and Cervidae. LTR divergence dating indicated that the integration of these ERVs occurred after the divergence of their respective host lineages, providing strong evidence for cross-species transmission events. This study not only deepens our understanding of red panda ERV classification but also provides a more comprehensive foundation for analyzing cross-species transmission events in mammalian retroviral evolution and offers a new perspective on ERV diversity and evolutionary mechanisms.

**IMPORTANCE:**

This study reports the discovery of complete endogenous retrovirus (ERV) sequences in the genome of the endangered red panda. We have annotated the genomic structures of these ERVs, which significantly enhance our understanding of their genetic composition and evolutionary history. Furthermore, by constructing host phylogenetic trees and performing LTR divergence dating, we identified trans-species transmission events of ERVs, shedding new light on the evolutionary interactions between ERVs and their hosts. These findings contribute to a broader understanding of microbial and viral diversity in the context of wildlife health and conservation, providing critical information to the scientific community.

## INTRODUCTION

Endogenous retroviruses (ERVs) are genetic elements capable of transcribing viral RNA genomes into DNA through reverse transcription and integrating them into the host genome (1). When the integration occurs in germ cells, ERV sequences can be stably inherited through host genome replication and vertically transmitted to offspring via Mendelian inheritance (2–4). ERVs typically consist of 5′ long terminal repeats (5′ LTRs), primer binding site (PBS), group-specific antigen (Gag), protease (Pro), polymerase (Pol), envelope (Env), polypurine tract (PPT), and 3′ long terminal repeats (3′ LTRs) (5). Among these, *gag*, *pro*, *pol*, and *env* are the key functional genes: *gag* encodes core proteins such as the capsid (6), matrix, and nucleocapsid (7); *pro* encodes the viral protease; *pol* encodes reverse transcriptase (RT) and integrase (INT); and *env* encodes the surface membrane protein (8) and transmembrane protein of retroviruses (9, 10). Current literature proposes multiple classification schemes for ERVs. In summary, ERVs have been categorized into three major classes: Class I (Gammaretrovirus/Epsilonretrovirus-like), Class II (Betaretrovirus-like), and Class III (Spumaretrovirus-like), based on phylogenetic analyses of conserved pol gene regions. Additionally, ERV classes are subdivided into several groups according to the sequence similarity of their primer binding site, which corresponds to tRNA complementary sequences (11).

The red panda belongs to the family Ailuridae within the order Carnivora. It is a small, endangered mammal that inhabits the eastern regions of Asia (8). As a rare and ancient animal species, the physiological characteristics and ecological habits of the red panda have been extensively studied; however, our understanding of its genome remains limited. Endogenous retroviruses have been found to be ubiquitous in vertebrate genomes, constituting a significant component of their genetic makeup (12). As a group within vertebrates, mammals have been reported to include retroviral sequences in more than 40% of their genomes (13). Nevertheless, to date, the existence and diversity of ERVs in the genome of the red panda are unknown. This study aims to analyze the ERVs in the red panda genome using comprehensive bioinformatics methods and phylogenetic analysis, validate the endogenous retroviruses present in red pandas, and discover the diversity of red panda ERVs and their relationships with other species. Ultimately, this research endeavors to provide new insights into the evolutionary history and genomic evolution of the red panda, while also expanding the understanding of the significance of ERVs in the field of biology.

## MATERIALS AND METHODS

### Identification of ERVs in the genome

This study utilized the red panda genome data (GCA_002007465.1) downloaded from NCBI, which is derived from a high-quality genome assembly as described in previous studies (14). We employed the blastx command within the Diamond alignment software to compare the genome files against the RetroFull protein database, with the *E*-value threshold set at 1E-18 (15). This approach aimed to preliminarily identify sequences within the red panda genome that potentially represent ERVs (Table S1). The RetroFull protein database contains 8,726 protein sequences about the main structural regions (Gag, Pro, Pol, and Env) of ERVs, serving as strong support for ERV alignments (Table S2). Among the screened sequence fragments, significant hits were filtered based on over 30% sequence identity and a length greater than 500 bp.

### Genomic annotation of ERVs

The filtered hit sequences were imported into Geneious Prime (version 2019.2.3) for examination of the distribution of open reading frames (ORFs). Sequences with longer ORFs were then traced back to their corresponding positions in the genome, and approximately 20,000 bp sequences flanking these ORFs were extracted for the search of complete ERVs. LTR_finder (16) was utilized to detect paired long terminal repeats within these extracted sequences. Simultaneously, the BLASTp tool (17) from the NCBI database was employed to search and annotate whether the complete ORFs in the sequences are potential ERVs, with the criterion of ORFs greater than 300 bp. For confirmed ERV ORFs, CD-Search against the CDD (https://www.ibs.renlab.org/) was used to identify conserved domains within them (7). To confirm the authenticity and copy number of the identified ERVs, we applied a strategy inspired by published research (18) and conducted BLASTn searches against the NCBI whole-genome shotgun contigs (WGS) database for all complete ERVs (19). Finally, the detailed structure of the full-length sequences was illustrated using the IBS2.0 online platform (https://www.ibs.renlab.org/) (20).

### Phylogenetic analysis

To better analyze the endogenous retroviral sequences obtained from the red panda genome, we selected a range of reference sequences, including those from seven distinct ERV genera and unclassified ERV sequences, to construct phylogenetic trees. RT, GAG, POL, and ENV protein sequences were aligned using MAFFT 7.526 (21). Poorly aligned regions (such as longer gaps resulting from the alignment process as well as unrecognized sequences present in the original genomic data) were removed using Gblocks (http://www.phylogeny.fr/) (6), and the alignments were manually confirmed with MEGA X (22). The best-fit models were estimated by the ModelFinder program of IQ-Tree (RT: rtREV + G4, GAG: VT + I + G4, POL: rtREV + F + R4, and ENV: WAG + F + I + G4). The phylogenetic trees for these protein sequences were inferred by the maximum likelihood method in IQ-Tree (23), with 1,000 bootstrap replications to evaluate the robustness of the nodes. The phylogenetic trees were viewed and annotated using FigTree version 1.4.4 (24), and finally, the trees were optimized using the iTOL online website (https://itol.embl.de/) (25).

### Molecular dating of ERVs

The integration time of ERVs can be estimated using the relationship *T* = (*D*/*R*)/2, where *T* represents the integration time (in million years, MYA), *D* is the nucleotide divergence between paired LTRs, and *R* is the neutral substitution rate per site per year (26, 27). Due to the absence of a species-specific substitution rate for the red panda, we applied the average mammalian neutral genomic substitution rate of 2.2 × 10^−9^ substitutions per site per year (28). LTRs with nucleotide divergence exceeding 10% were excluded from this analysis, including the paired LTRs of ERV-Beta.2-Aful, as their divergence exceeded this threshold.

## RESULTS

### Discovery and classification of retroviral elements in *Ailurus*

**Fig 1:**
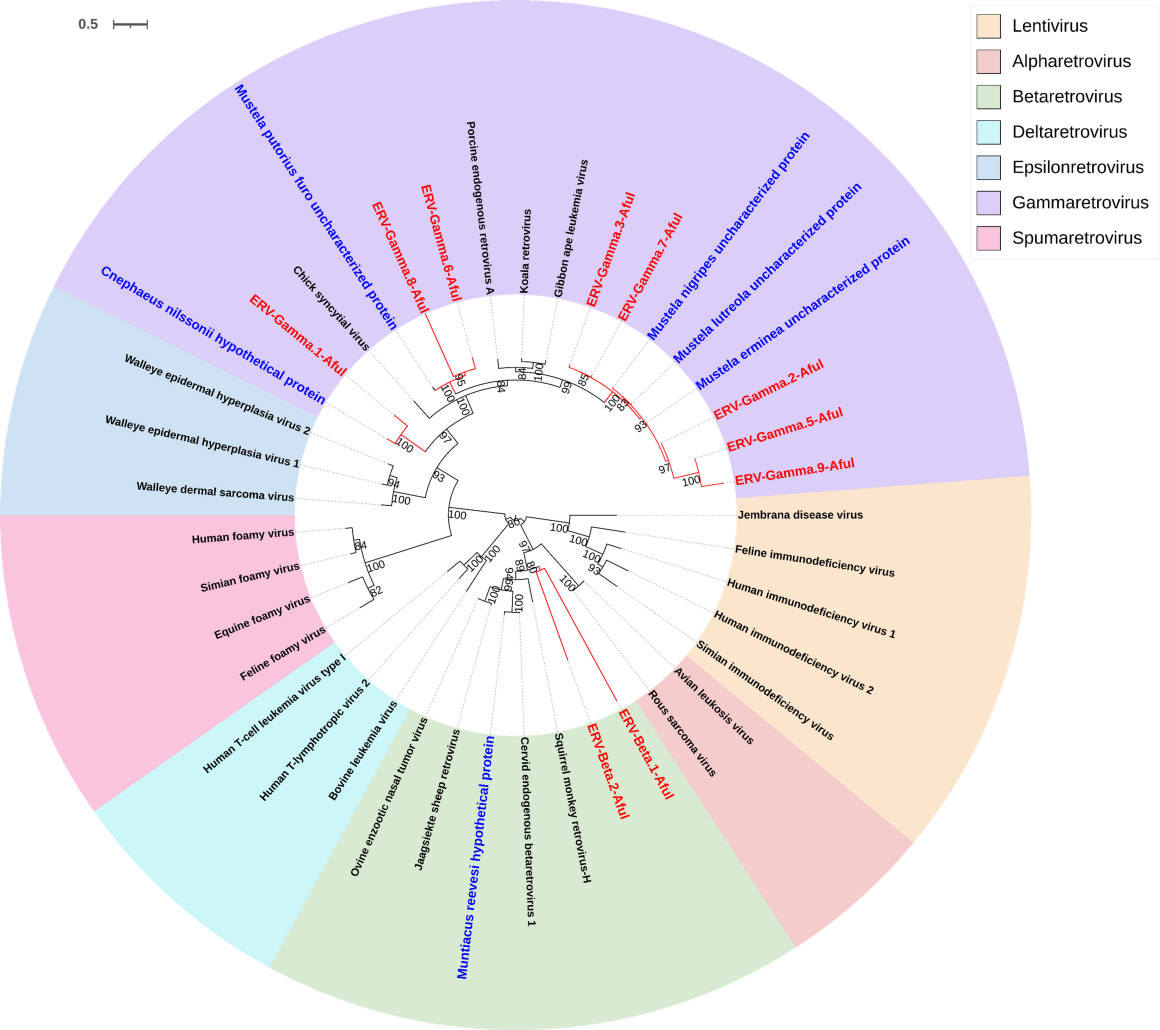
Unrooted phylogeny of red panda ERVs and other retroviruses. The tree was inferred from RT protein alignment. The node robustness was evaluated through ultrafast bootstrap with 1,000 replicates. The newly identified red panda ERVs are labeled in red. The scale bar indicates the number of base changes at each site.

Through comparison with the RetroFull protein database, we identified endogenous retroviral elements in the red panda genome and subsequently screened out 11 relatively intact ERVs. Based on representative reverse transcriptase sequences derived from retroviruses, we constructed a phylogenetic tree incorporating 10 of these ERVs (excluding one due to the absence of its RT sequence). The tree demonstrated that the retroviruses identified within the red panda genome belonged to two well-supported genera: Gammaretrovirus and Betaretrovirus. This classification confirmed their identity as authentic red panda ERVs, among the seven recognized retroviral genera that also include Epsilonretrovirus, Spumaretrovirus, Alpharetrovirus, Deltaretrovirus, and Lentivirus (Fig. 1). According to the naming conventions for ERVs (29), the topology of the phylogenetic tree, and the sequential order of the ERVs in their original genomic loci in the red panda, the identified ERVs were named as follows: ERV-Gamma.n-Aful (*n* = 1–9) and ERV-Beta.n-Aful (*n* = 1–2) (Table 1). It is worth noting that, due to the absence of its RT component, ERV-Gamma.4-Aful was classified as a member of the Gammaretrovirus genus in subsequent phylogenetic analyses of the GAG, POL, and ENV sequences.

**TABLE 1:** Distribution of 11 ERVs on the red panda genome^*a*^

Name	Distribution		Length (bp)	LTR region similarity
	Location (on scaffold)	Location (bp)		
ERV-Gamma.1-Aful	LNAC01000019.1	2,124,963–2,132,462	7,500	0.972
ERV-Gamma.2-Aful	LNAC01000230.1	2,255,945–2,263,422	7,478	–
ERV-Gamma.3-Aful	LNAC01000369.1	1,575,317–1,581,692	6,376	–
ERV-Gamma.4-Aful	LNAC01000793.1	326,765–332,446	5,682	–
ERV-Gamma.5-Aful	LNAC01000901.1	194,043–202,315	8,273	–
ERV-Gamma.6-Aful	LNAC01001344.1	32,044–39,936	7,893	0.97
ERV-Gamma.7-Aful	LNAC01001384.1	133,637–140,487	6,851	0.973
ERV-Gamma.8-Aful	LNAC01001395.1	173,803–181,620	7,818	0.94
ERV-Gamma.9-Aful	LNAC01001561.1	18,697–25,652	6,956	–
ERV-Beta.1-Aful	LNAC01000031.1	2,362,551–2,368,857	6,307	0.935
ERV-Beta.2-Aful	LNAC01001339.1	67,540–73,772	6,233	0.98

^*a* *–*^ , absence of LTR.

### Characterization of ERVs in the *Ailurus* genome

**Fig 2:**
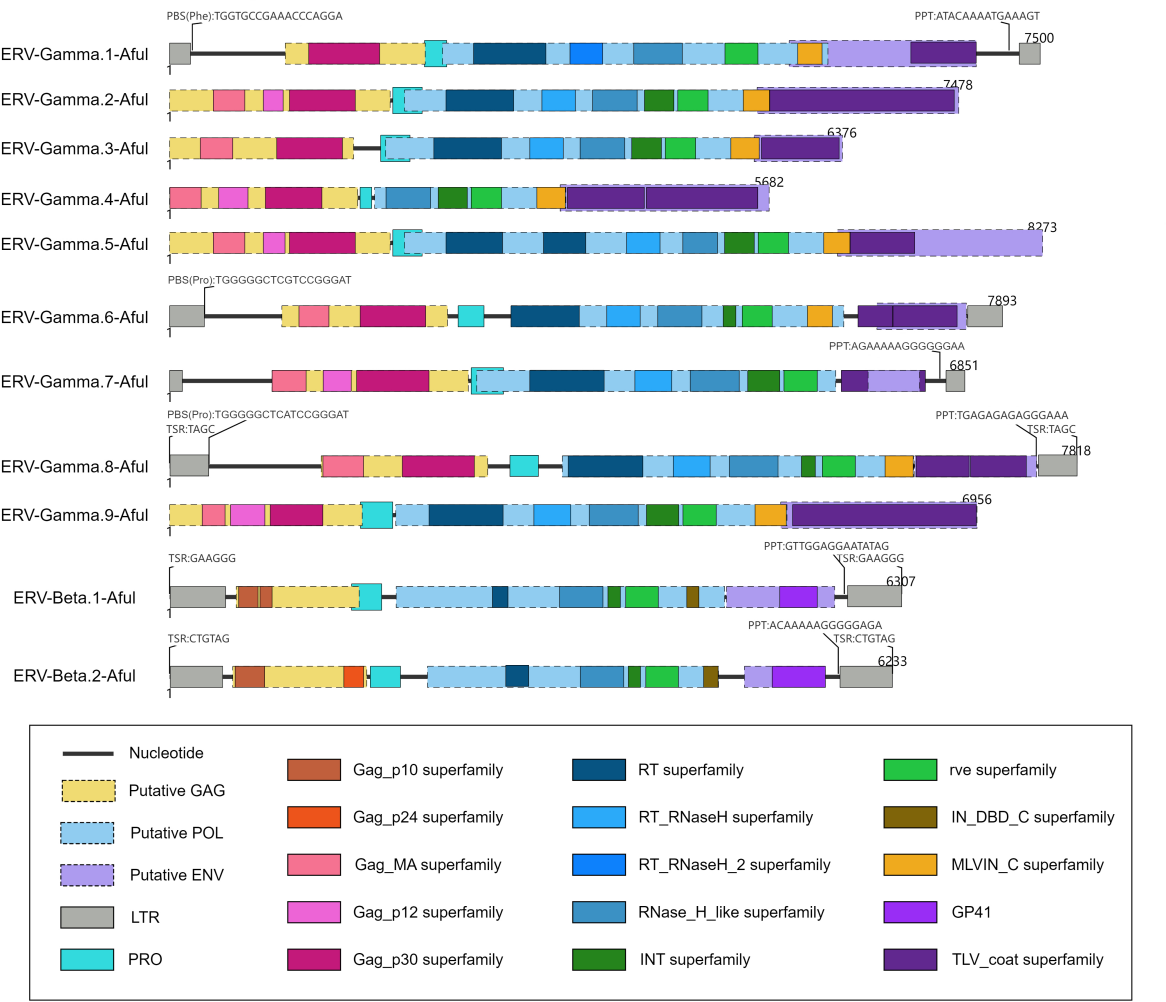
Genomic organization of red panda ERVs. The genomes of red panda ERVs are drawn to scale using lines and boxes. Putative ORFs are shown in dotted boxes and were used to determine viral coding regions. The predicted domains or regions that encode conserved proteins are represented by colored boxes. GAG, group-specific antigen gene; POL, polymerase gene; and ENV, envelope gene.

Among the 11 ERVs identified in the red panda genome, six were confirmed to be complete based on the presence of paired LTRs within their flanking sequences (Table 1). To further characterize their genomic structural features, we annotated the PBS, PPT, and target site repeat (Fig. 2). Notably, the PBS sequence of ERV-Gamma.6-Aful (Pro) was identical to that of mammalian retroviruses, while the PBS sequences of ERV-Gamma.1-Aful (Phe) and ERV-Gamma.8-Aful (Pro) both exhibited a single base mutation (30). Through analysis of their conserved structural domains, we found that all 11 ERVs exhibited typical retroviral genome structures, including three core genes: gag, pol, and env (Fig. 2). Additionally, the conventional conserved domains of retroviruses, including the RT superfamily, the RT_RNaseH superfamily, the RNase_H_like superfamily, the INT superfamily, the integrase core (rve) superfamily, the MLVIN_C superfamily, and the IN_DBD_C superfamily, were widely distributed in the genome of the red panda. However, not every ERV identified contained all these domains, as they exhibited internal deletions. The occurrence of internal gene deletions in these ERVs strongly suggested significant structural variations and potential functional degradation that may have occurred during their evolution within red pandas.

### Phylogenetic analysis of *Ailurus* ERVs

**Fig 3:**
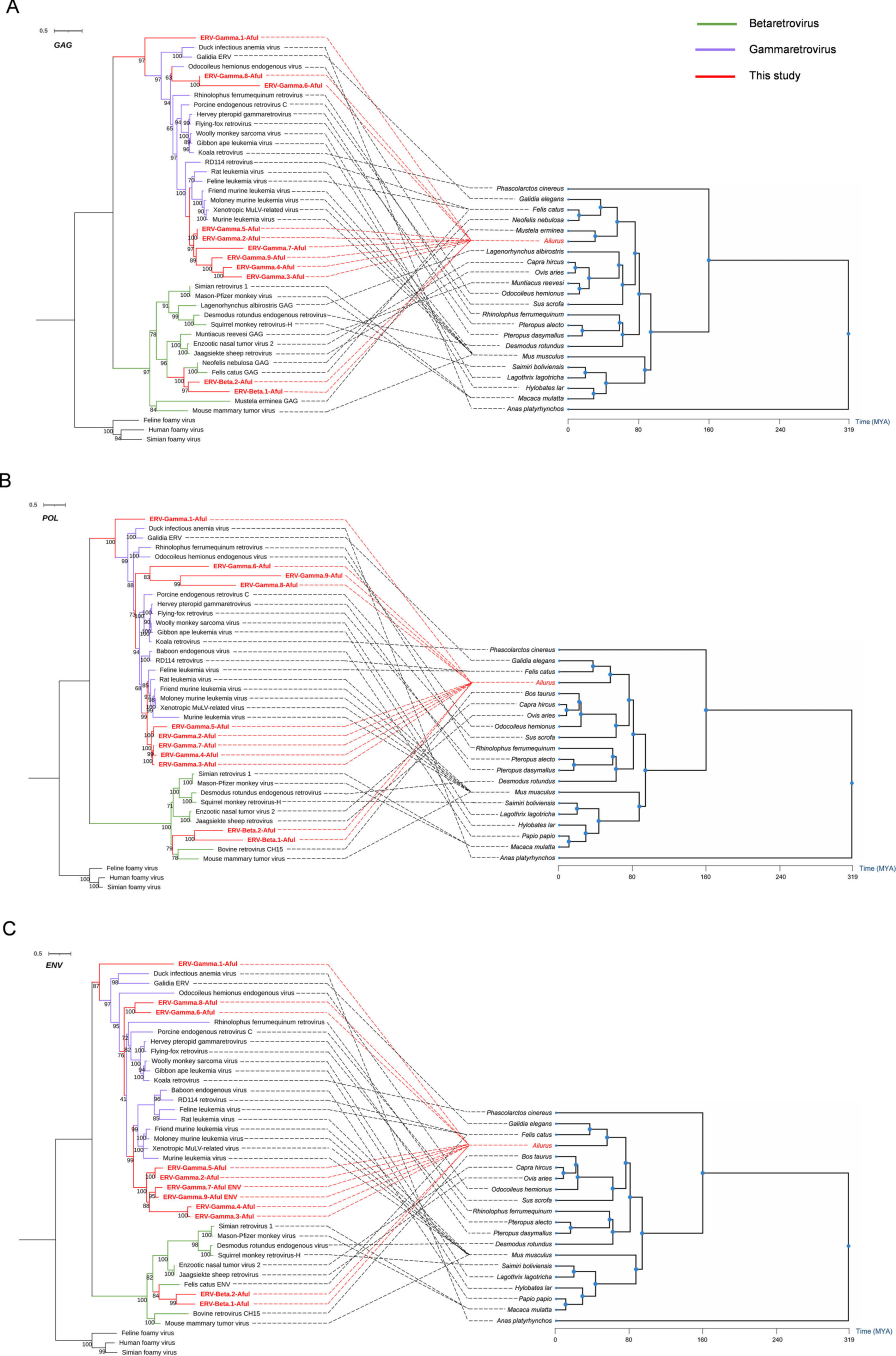
Phylogenetic trees of red panda ERVs and their hosts. The trees were inferred using amino acid sequences of GAG (A, left), POL (B, left), and ENV (C, left). The phylogenetic trees of hosts (A, right; B, right; and C, right) were generated by using TimeTree (http://timetree.org/). All the trees were rooted by foamy retroviruses. The newly identified red panda ERVs are labeled in red. Green and purple indicate Betaretrovirus and Spumaretrovirus, respectively. The scale bar indicates the number of amino acid changes per site.

To investigate the relationship between red panda ERVs and other known exogenous and endogenous mammalian ERVs, we constructed phylogenetic trees based on conserved amino acid sequences of the GAG, POL, and ENV regions, respectively. Phylogenetic analysis revealed that ERV-Gamma.2-Aful, ERV-Gamma.3-Aful, ERV-Gamma.4-Aful, ERV-Gamma.5-Aful, and ERV-Gamma.7-Aful consistently formed a robust clade, with bootstrap values exceeding 95% across all three regions (Fig. 3), indicating their high homology and shared common ancestry. Similarly, ERV-Beta.1-Aful and ERV-Beta.2-Aful consistently clustered together across all three regions, further supporting their close evolutionary relationship. These results underscore the evolutionary conservation and common genetic origin of red panda ERVs.

Furthermore, the phylogenetic trees revealed that the red panda ERVs exhibited close phylogenetic relationships with specific exogenous retroviruses across all three genomic regions. The clade formed by ERV-Gamma.2-Aful, ERV-Gamma.3-Aful, ERV-Gamma.4-Aful, ERV-Gamma.5-Aful, and ERV-Gamma.7-Aful consistently clustered with several murine leukemia viruses from the Muridae family, suggesting their origin from a common ancestral retrovirus. Similarly, the ERV-Beta.1-Aful and ERV-Beta.2-Aful clades demonstrated stable associations with uncharacterized feline retroviruses in both GAG and ENV trees, further supporting shared ancestral origins. Notably, the topologies of the GAG, POL, and ENV phylogenetic trees were not entirely consistent. For instance, ERV-Gamma.9-Aful clustered with murine ERVs in the GAG and ENV phylogenetic trees but exhibited closer affinity to cervid ERVs in the POL tree, indicating that different genes of ERVs may possess distinct evolutionary trajectories. The observed inconsistency between red panda ERVs and their host species suggested potential cross-species transmission events, contributing to the genetic diversity and host range of red panda ERVs.

In summary, red panda ERVs have not only maintained a high degree of conservation during evolution but have also developed complex genetic diversity through interactions with exogenous retroviruses. They likely originated from ancient mammalian retroviral insertions, followed by cross-species transmission events that introduced them into the red panda lineage, where they underwent lineage-specific fixation during evolutionary divergence. These findings have provided critical insights into the evolutionary history, cross-species transmission mechanisms, and functional roles of red panda ERVs within the host genome.

### Estimation of the insertion time of *Ailurus* ERVs

A divergence-based dating method for paired LTRs was employed to estimate the insertion times of different lineages of red panda ERVs (26, 27), aiming to further elucidate their evolutionary history. To ensure accuracy, only ERVs with complete LTR sequences were included in the analysis, specifically ERV-Gamma.1-Aful, ERV-Gamma.6-Aful, ERV-Gamma.7-Aful, ERV-Gamma.8-Aful, and ERV-Beta.1-Aful sequences (Table S3). The results indicated that the insertion time of the ERV-Gamma-Aful lineage ranged from 6.36 to 14.41 million years ago (MYA), while that of the ERV-Beta-Aful lineage was traced back to 12.86 MYA. The high similarity between paired LTRs and the estimated insertion time suggested that red panda ERVs represented a relatively young group of ERVs, indicating recent integration into the red panda genome.

## DISCUSSION

In this study, we conducted a comprehensive analysis of endogenous retroviral elements in the red panda genome. By aligning with the RetroFull protein database, we successfully identified 11 ERV sequences, with an average length of 7,000 bp. Based on the presence of paired LTRs, six of these sequences were confirmed as intact ERVs. The remaining five ERVs, although lacking LTRs, exhibited relatively intact internal structures, including the core genes *gag*, *pol*, and *env*, albeit with occasional internal deletions. This structural variation may be attributed to the continuous accumulation of genetic mutations and frequent recombination events during the long evolutionary history, leading to varying degrees of structural degeneration in some ERVs.

Further phylogenetic analysis revealed the evolutionary complexity of ERVs in the red panda genome. Based on reverse transcriptase sequences, the identified ERVs were classified into two well-supported genera: Gammaretrovirus and Betaretrovirus. Notably, the majority of the ERVs clustered within Gammaretrovirus, while a smaller subset was assigned to Betaretrovirus. ERV-Gamma.4-Aful, although lacking an RT domain, was classified as Gammaretrovirus based on other conserved domains, suggesting that recombination events shaped the evolutionary trajectories of these elements. This finding aligns with the broader understanding of ERVs as dynamic components of the genome, subject to both vertical inheritance and horizontal transfer processes.

Phylogenetic trees constructed from the conserved amino acid sequences of the GAG, POL, and ENV regions further revealed the evolutionary relationships between red panda ERVs and those from other species. The consistent clustering of ERV-Gamma.2-Aful, ERV-Gamma.3-Aful, ERV-Gamma.4-Aful, ERV-Gamma.5-Aful, and ERV-Gamma.7-Aful across all three regions, with high bootstrap support, indicated a shared common ancestry and evolutionary conservation. The topologies of the GAG, POL, and ENV phylogenetic trees were not entirely consistent, suggesting that different genes of ERVs may exhibit distinct evolutionary trajectories. The close phylogenetic relationships between red panda ERVs and certain exogenous retroviruses, such as murine ERVs and feline ERVs, imply that cross-species transmission has contributed to the genetic diversity of these elements. LTR divergence dating revealed that the integration of red panda ERVs occurred after the divergence of host lineages, providing strong evidence for cross-species acquisition. This finding aligns with previous studies reporting population expansion in the red panda during its historical evolution (31), further supporting the dynamic nature of ERV integration and evolution within this species.

In conclusion, our study reports the presence of relatively intact ERVs in the genome of the red panda, thereby enriching the existing database of ERVs in mammals. The phylogenetic analysis conducted herein investigates the host range of red panda ERVs and reveals potential associations between these ERVs and those of other species, suggesting that red panda ERVs may have originated from cross-species transmission events. LTR divergence dating further supports these cross-species transmission events. Collectively, these findings not only highlight the dynamic interplay between viral evolution and host genomic adaptation but also provide critical temporal and phylogenetic evidence for understanding ERV-mediated genetic diversification in mammals.

## SUPPLEMENTAL MATERIAL

Table S1 spectrum.00024-25-s0001.xlsx ERV elements from Red Panda genome aligned with RetroFull database

Table S2 spectrum.00024-25-s0002.xlsx RetroFull database sequence.

Table S3 spectrum.00024-25-s0003.xlsx Estimation of the insertion time of ERVs.

ASM does not own the copyrights to Supplemental Material that may be linked to, or accessed through, an article. The authors have granted ASM a non-exclusive, world-wide license to publish the Supplemental Material files. Please contact the corresponding author directly for reuse.
